# Two Cases of Splanchnic Nerve Block With Epidural Anesthesia in Patients Unable to Maintain Prone Position Due to Pancreatic Pain

**DOI:** 10.1089/pmr.2023.0031

**Published:** 2023-09-28

**Authors:** Shunya Sekiguchi, Yusuke Ishida, Mikiko Tomino, Kiyoshige Ohseto

**Affiliations:** Department of Anesthesiology, Tokyo Medical University Hospital, Shinjuku-ku, Tokyo, Japan.

**Keywords:** epidural anesthesia, pancreatic cancer, prone position, splanchnic nerve block

## Abstract

Splanchnic nerve block is considered to be effective for abdominal visceral pain, and is performed for the purpose of controlling abdominal pain and back pain caused by upper abdominal cancer. The patients in this case report were candidates for splanchnic nerve block owing to cancer-associated pain. However, because they could not assume the prone position that is required for the block owing to their pain, combined epidural anesthesia was used, resulting in successful implementation of the splanchnic nerve block. Patients who are candidates for splanchnic nerve block often have advanced cancer, and it is hence often difficult to secure and maintain the position required for the procedure owing to their severe pain. The two patients presented here suggest the possibility that epidural anesthesia might be useful as an adjunct in such cases.

## Introduction

The celiac plexus lies anterior to aorta at the level of the first lumber vertebra. Splanchnic nerves are paired nerves arising from the thoracic sympathetic trunk (ganglia 5–12) which pierce the crura of the diaphragm at the T11 and T12 levels to join the celiac ganglion. Splanchnic nerve block is considered to be effective for visceral pain in the upper abdomen and is performed in the prone position under computed tomography (CT) guidance. In this report, we present two cases in which the addition of epidural anesthesia facilitated the successful administration of splanchnic nerve block in patients who were considered to be suitable for the procedure owing to their cancer-associated pain, but they were unable to assume the position required for the block, owing to their pain. We obtained written informed consent from the patients to publish this case report.

## Case Presentation

**FIG. 1.**
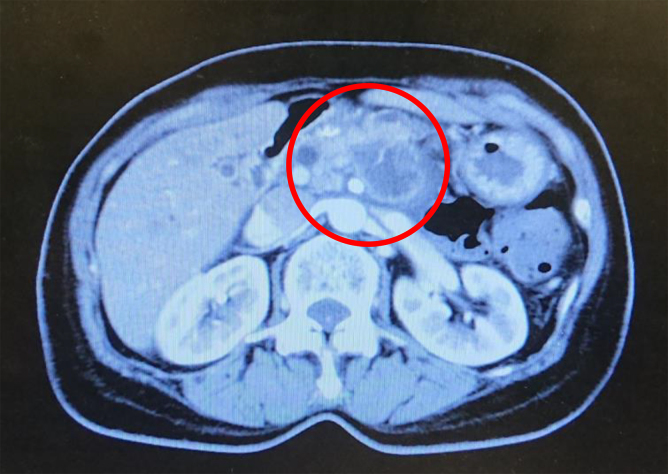
Abdominal computed tomography image of the first patient. A tumor of ∼36 mm in the main body of the pancreas (red circle), which appeared to have invaded the splenic artery, celiac artery, and portal vein was displayed.

The first case was a woman in her sixties who was diagnosed with pancreatic cancer and had epigastric and back pains, and she was referred to our department for pain control. The prescription included a fentanyl transdermal patch (167 mcg/h), loxoprofen sodium hydrate (180 mg/day), and oxycodone hydrochloride hydrate (45 mg/day). The patient reported symptoms of stabbing pain in the epigastric region and dull pain in the back, and sometimes complained of unbearable pain. She had no appetite and also had sleep disturbances. She had a numerical rating scale (NRS) score of 10/10 for pain, a Self-Rating Depression Scale (SDS) score of 43/80, a Kessler Psychological Distress Scale (K6) score of 23/24, and an EuroQol 5 Dimension (EQ-5D) score of 0.594. Abdominal CT showed a tumor of ∼36 mm in the body of the pancreas, with possible infiltration into the splenic artery, celiac artery, and portal vein (Fig. 1). After considering the case, the patient was scheduled to undergo a splanchnic nerve block under CT guidance for pain control 22 days after the initial consultation at our department. When attempting to maintain the prone position for the CT-guided splanchnic nerve block, the patient had difficultly sustaining the position owing to her abdominal pain. Therefore, to relieve the pain caused by maintaining the prone position, an epidural puncture was performed at the T10/11 level to insert a catheter for the purpose of pain relief, and 4 mL of 1% lidocaine was injected through the catheter. The patient experienced pain relief a few minutes after lidocaine administration, but at the same time, a decrease in blood pressure (90/40 mmHg) occurred. Therefore, the patient's legs were elevated, and 0.1 mg of phenylephrine was administered intravenously, followed by an infusion of 500 mL of 6% hydroxyethyl starch, resulting in an improvement in blood pressure (140/80 mmHg). The patient was then placed in a prone position, and the CT-guided splanchnic nerve block was started. During the block procedure, there were no complications, such as a decrease in blood pressure, and the procedure was performed safely.

**FIG. 2.**
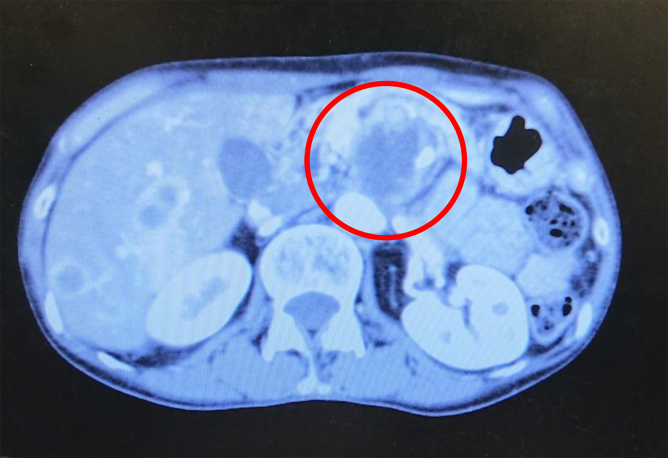
Abdominal computed tomography image of the second patient. A tumor of ∼27 mm in the pancreatic head (red circle), and multiple metastases in the liver were displayed.

The second case occurred in a woman in her fifties with pancreatic cancer. She complained of severe pain in the epigastric and back regions, and she was referred to our department for pain control. She was taking oxycodone hydrochloride hydrate (30 mg/day) and loxoprofen sodium hydrate (189 mg/day). She had no appetite, but did not have any sleep disturbances. Her NRS was 6/10, SDS was 45/80, K6 was 3/24, and EQ-5D was 0.228. Abdominal CT showed a tumor of about 27 mm in the pancreatic head, and multiple metastases were observed in the liver (Fig. 2). The day after her visit to our department, it was decided that a splanchnic nerve block should be performed for the pain. This patient also had difficulty maintaining the prone position owing to the pain, so it was decided that epidural anesthesia would be performed before the CT-guided splanchnic nerve block. An epidural catheter was placed via a puncture at the T9/10 level, and subsequently, 4 mL of 1% lidocaine was administered through the catheter. The patient was placed in the prone position after the epidural anesthesia was performed, and the splanchnic nerve block was started. In this case, the patient's blood pressure remained stable and did not show any significant decrease after the epidural anesthesia was administered. Furthermore, no major complications were observed during the visceral nerve block procedure.

## Discussion

**FIG. 3.**
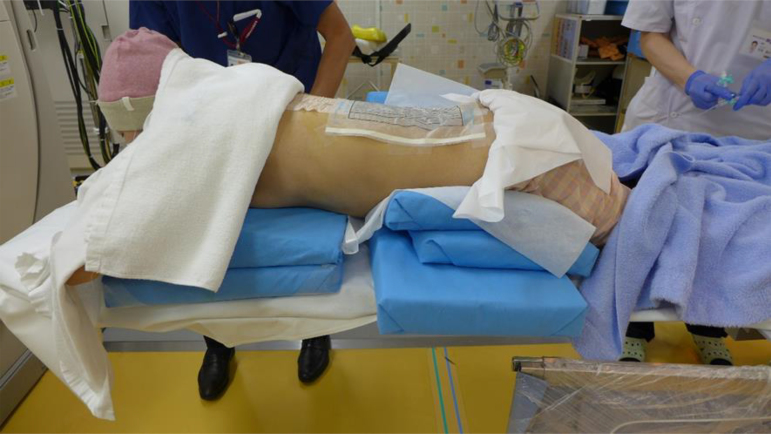
Position of the patient during the splanchnic nerve block. The patient is placed in a prone position, and this position must be maintained for a substantial amount of time.

Splanchnic nerve block is indicated for upper abdominal pain and back pain caused by cancer of the upper abdominal organs.^1–3^ The patients of both cases presented in this report had poor pain control with opioids and experienced visceral pain owing to pancreatic cancer. Therefore, splanchnic nerve block was felt indicated for both patients. In general, when performing a splanchnic nerve block, the patient is placed in the prone position^4,5^ (Fig. 3). We performed splanchnic nerve block under CT guidance for both patients, but the procedure can also be performed under X-ray fluoroscopy guidance, which also requires the prone position.^6^ For both techniques, it is necessary to maintain the prone position for a substantial amount of time during the procedure. However, both of our patients found it difficult to maintain the prone position owing to their pain. Therefore, we combined epidural block to relieve the pain in the region of T6-10 first.

By combining the procedure with epidural block, the patients were able to maintain the prone position for the required time, leading to an effective and successful splanchnic nerve block. In addition, insertion of a catheter made it possible to administer local anesthetics again during the procedure if the pain returned. In the first patient, a decrease in blood pressure occurred after the epidural anesthesia. This appeared to be caused by sympathetic nerve blockage owing to the epidural anesthesia and to being in a mild dehydrated state as a result of abstinence from eating and drinking before the procedure. In the second patient, no complications caused by the epidural anesthesia were observed.

In general, when splanchnic nerve block is indicated for a patient with pancreatic cancer, it is expected that the cancer has progressed to some extent and that the patient's general condition is unfavorable; therefore, when performing epidural anesthesia, extra caution is required for possible side effects. In addition, attention must be paid to the concentration and dosage of local anesthetics. In these two cases, the initial use of epidural anesthesia enabled patients to maintain the prone position during the procedure.

However, if epidural anesthesia cannot be performed for some reason, such as spinal degeneration, increased intracranial pressure, or extreme hypovolemia, another option for relieving abdominal and back pain is a transverse abdominis plane (TAP) block. A TAP block is generally considered to provide somatic analgesia, but without any effect on visceral pain. Importantly, there have been several reports that TAP blocks were effective in treating visceral pain.^7,8^

## Conclusion

Based on the findings of the two cases presented in this report, the combined use of epidural anesthesia can be considered to be helpful in managing the difficulties associated with patients maintaining the prone position during splanchnic nerve block. However, precautions should be taken to prevent complications, such as hypotension during administration of epidural/spinal anesthesia.

## Abbreviations Used

CT: computed tomography
EQ-5D: EuroQol 5 Dimension
K6: Kessler Psychological Distress Scale
NRS: numerical rating scale
SDS: Self-Rating Depression Scale
TAP: transverse abdominis plane
